# Surgery

**Published:** 1858-01

**Authors:** 


### SURGERY.

1. THE ESCHAROTIC TREATMEMT OF CANCER. By *James Syme*, Professor of Clinical Surgery in the University of Edinburgh.—The subject of cancer has been so mystified by the misrepresentations of unscrupulous adventurers, that any attempt to re-establish its treatment upon sound principles may seem a hopeless undertaking; but I nevertheless beg to offer the following observations, in the hope that they will still be of service where rapacity and credulity have not entirely superseded honesty and common sense. It is not surprising that the dread of cutting, and the bad success of operations, too frequently performed under unfavorable circumstances, should have predisposed to the ready reception of any new proposal for the remedy of a disease so formidable; and, accordingly, the employment of variously prepared caustics for this purpose has, during the last twenty or thirty years, afforded a fruitful field for quackery without and also within the profession. For, whatever may be the sentiments or laws of medical corporations, I shall always hold, that the worst form of quackery is not practicing without a diploma, but using secret remedies, and pandering, by false pretences, to the capricious folly of credulous patients. It could hardly, however, have been anticipated that the medical officers of a metropolitan hospital would so far forget the dictates of professional honor as not only to harbor in their institution a secret cancer-curer, but, by continuing to do so month after month, sanction the delusion that he possessed some beneficial means of treatment unknown to their brethren. Yet the surgeons of the Middlesex Hospital have exposed themselves to this serious charge. They can hardly deny the former part of it; and with regard to the latter, must either plead guilty or profess belief that the juice of *Sanguinaria Canadensis* possesses more active powers than the gilding of a pill. In their anxiety to escape from the horns of this painful dilemma, they now admit that "the vegetable ingredient is practically inert," that the caustic is an old one, and that there is nothing new in the practice of their protégé except daily making incisions and stuffing the wounds with escharotic paste,—a proceeding utterly opposed to the established principles of surgery, and eminently calculated to produce the most disastrous consequences, but which, instead of indignantly condemning, they commend as deserving of general adoption, and as entitling its author to public gratitude. The hollow pretensions of secrecy having been thus endorsed and published with the authority of a metropolitan hospital, it is requisite for the protection of sufferers from cancer that the principles of its proper treatment should, so far as possible, be no less extensively diffused.

It being admitted, by universal consent, that the various forms of disease comprehended under the titles of cancer and carcinoma are not remediable except through removal of the morbid part, the only room for question that remains in regard to their treatment is limited to the choice of means for this purpose. There can be no doubt that excision, if performed under chloroform, affords not only the most speedy and effectual, but also the least painful, mode of extirpating the disease, so far as its extent can be recognized by sensible characters. On the other hand, it is alleged that through the use of caustic a more lasting protection may be obtained against the danger of relapse; and if such were really the case, there could be no hesitation in preferring the latter means. But, unfortunately, from being chiefly used empirically, they are supported by evidence of a very questionable character. For, in the first place, patients who confide their treatment to irregular practitioners, are naturally unwilling to admit that they have received no benefit or suffered damage from venturing upon this course. They are, consequently, very ready to be persuaded that good has been done, and when the expectations thus excited prove to be fallacious, no less slow to confess their disappointment. The wonderful histories of cures, therefore, so frequently put in circulation on the ground of such sanguine anticipations, are seldom counteracted by a knowledge of the issue, however disastrous it may have been. Thus, an impudent quack—the Middlesex surgeons call him a "gentleman"—industriously distributed penny puffs, red, green, and yellow, of which the most prominent feature was an affidavit sworn before the Lord Provost of Glasgow, by a man who had been an out-patient at the hospital here, under my care, that he was cured by the said quack, although he was not so, and died soon afterward of the disease. Now, this poor creature doubtless believed the assurance of that respectable person, and probably signed his declaration from the amiable motive of leading fellow-sufferers in a right direction; but having taken this unwary step, could not retrace it, when taught by sad experience that he had been miserably deceived.

Then, again, there are so many diseased conditions apt to be regarded as incurable, although not really so, that the most careful discrimination is required to prevent their successful treatment from being erroneously assumed as ground for belief in the curability of cancer. But the empirical practitioner is neither able nor willing to make such distinctions, and, so far from endeavoring to dismiss the unfounded apprehensions of a patient, will always be anxious to cherish and increase them, in order to enhance the value of his pretended services. Thus, if a tumor of the breast, supposed to be carcinomatous, should be a serous cyst requiring merely evacuation and irritation of the surface, or a fibrous growth removable without further disturbance of the gland, or a chronic abscess, or even nothing more than that simple engorgement and painful state so common in females whose health is out of order, the quack will not vary his practice or scruple to make the unfortunate patient pass through all the horrors of a prolonged escharotic treatment. Indeed, I once saw a poor woman who had both of her breasts destroyed by caustic, although there was distinct evidence that neither of them had been at all diseased.

In regard to the comparative time and danger of cutting and caustic, there can be no doubt that, so far as the first of these points is concerned, the former mode of relief is greatly preferable; while, as to the last one, there does not seem to be much difference between the two. Unless, therefore, it can be shown that the escharotic treatment is more complete and permanent in its effect than excision, it will be difficult to discover any good reason for abandoning the knife, or complicating it with the addition of caustic. The surgeons of the Middlesex Hospital have published an account of the cases treated under their inspection, which, although evidently drawn up with the desire of presenting a favorable view, may, of course, be regarded as an authentic statement, and not as the mere trumpeting of quackery. They here relate that the process employed by their American coadjutor was to destroy the skin by nitric acid, then to make numerous incisions, and introduce chloride of zinc-paste into the wounds, which were repeated daily for from two to seven weeks, until the object appeared to be accomplished. They have not concealed the severe and protracted suffering endured by the patients subjected to this painful and tedious procedure, nor have they shrinked from confessing its dismal results. Forty-two cases of cancer or schirrous breast are recorded. Of these, five were considered unfit for treatment and declined; three left the hospital without any apparent local disease; and thirty-four still suffered from it in the form of enlarged glands, tubercles of the skin, or open sores. Anything more shocking than this it is impossible to imagine; and I sincerely hope that the conclusive testimony thus published, in a form, externally at least, well suited if not intended for the table of a drawing-room, may tend to counteract the present rage for escharotic treatment.

It has long been a settled principle in surgical practice, that malignant tumors or sores should be either allowed to remain free from disturbance or completely removed, since tampering with them by irritating applications is the most certain means of exciting disease in the lymphatic glands or other textures. But the procedure advocated by the Middlesex surgeons was the most extreme degree of deviation from this rule, since it kept the local disease, together with the patient's system, in a perpetual fret for many weeks; so that no one need be surprised at the effects, which, indeed, these gentlemen thus admit: "Nothing could be more disastrous than this case; and there is no reasonable doubt that the tumultuous increase of the disease was directly owing to the local treatment." If caustic is ever used for destroying malignant textures, it should, therefore, be of such power and so employed as to strike at once to the root of the evil, and I am able to suggest efficient means for this purpose.

Mons. Velpeau, in speaking of the caustic made by mixing sulphuric acid with saffron, expresses his persuasion that it would be the best of all escharotics except for its expense and the difficulty of confining its action within certain limits. It occurred to me that sawdust would supply the place of saffron, and my assistants at the hospital ingeniously devised the following effectual means of restraining the extent of action:—A solution of gutta percha in chloroform is applied to the skin for some distance round the part to be attacked; then a thick piece of the same material, with an aperture cut in it of the requisite size, and softened by exposure to heat, is pressed firmly so as to adhere everywhere to the surface thus prepared; a thin piece is next glued round the edge of the opening, so that, when supported by a stuffing of lint, it may form a wall inclosing the diseased part. Concentrated sulphuric acid, with about an equal weight of sawdust stirred into it, until the mixture assumes a homogeneous consistence equal to that of thin porridge, is lastly applied, in quantity proportioned to the extent of thickness concerned. In the first instance, as the pain is acute, opiates or chloroform may be used; but after a short while so little uneasiness is felt that the patient can easily allow the caustic to remain for ten or twelve hours, when it will be found that the whole diseased mass, though covered with skin and several inches in depth, has been reduced to a cinder, presenting the appearance of strongly compressed tow. Under poultices the slough separates in the course of days or weeks, according to its depth, and the sore then heals without any trouble. If, therefore, patients, from an unconquerable dread of cutting, should prefer the escharotic treatment, or if the circumstances on any other account should seem to render this method eligible, the procedure just described may be found useful.

In conclusion, I beg to offer the following principles or practical rules for the treatment of cancer:—

1. The treatment of cancer may be divided into curative and palliative.
2. The curative treatment should not be undertaken when the local disease is so seated or connected as to prevent its complete removal; when the lymphatic glands are affected; and when the patient's general health is deranged.

3. Removal may be accomplished by means of the knife, escharotics, and ligatures.

4. Of these means, in general the knife is best, and ligatures the worst.

5. Escharotics may be used with most advantage when the disease is superficial.

6. Escharotics, employed with a curative view, should always destroy the whole morbid part by one application.

7. The palliative treatment is generally best accomplished by means of soothing applications and attention to the general health.

8. When the local disease is very troublesome it may sometimes be relieved for a time by destruction of the morbid growth.

9. The best agent for this purpose, and also with a curative view, is concentrated sulphuric acid properly applied.—*Edinburgh Medical Journal*, Nov. 1857.

2. Excision of Joints.—[The most notable feature presented by operative surgery in Great Britain during the past few years, is the extent to which resection of the articular ends of the bones of the extremities has been carried. In the rage which at present seems to prevail in London for this formidable procedure, doubtless many patients have been made to suffer unnecessarily; but, on the other hand, we are equally sure that a larger number have been saved from far worse mutilation, or from death itself. The operation may, in deed, be looked upon as one of the great triumphs of modern surgical art; and as exhibiting in some measure its favorable results, the character of the cases to which it is adapted, and the high authority for its performance, we extract from recent numbers of the *Medical Times and Gazette* the following statistical report of the principal London hospitals for the second quarter of the present year.]

Case I.—St. Thomas's: Mr. Solly.—A man, aged twenty-seven, in very reduced health from disease of the knee-joint of a year's duration. Numerous sinuses existed, and there had been profuse discharge. The articular surfaces of the three bones were sawn away in each instance very superficially. The man afterward sank, death taking place about three weeks after the operation.

Case II.—St. Thomas's: Mr. Simon.—A man, aged forty-two, in very delicate health. Disease of the ankle-joint had existed for three years, and several sinuses existed. Excision of the articular surfaces of the tibia, fibula, and astragalus was performed, the latter bone being very extensively diseased. The patient sank, and died on the fourth day.

Case III.—St. Thomas's: Mr. Simon.—A man, aged forty, the subject of disease of the extremity of the humerus. A piece of necrosed bone had been removed some months previously, and on the present occasion a partial excision of the joint was performed, the outer condyle being wholly cut away. The ends of the other bones being sound, were allowed to remain. Recovered well, but the joint will probably be stiff.

CASE IV.—The Dreadnought: Mr. Tudor.—A Lascar, aged thirty, under care on account of disease of the left elbow-joint. Several sinuses existed, and he was much reduced in health. Excision of the lower fifth of the humerus, of the ulna below the cornoid process, and of the articular inch of the radius, was performed. It was the separation of the periosteum and the roughened state of the bones which rendered such an extensive resection needful. The man has done remarkably well, and the wound is now nearly healed. As yet there is not much power of voluntary movement.

CASE V.—St. Thomas's: Mr. South.—A boy, aged nine, under care on account of disease of the knee-joint, of strumous character, and of six months' standing. Resection of the articular ends of the bones on May 30. Favorable progress.

CASE VI.—St. Thomas's: Mr. Le Gros Clarke.—A boy, aged fifteen. Disease of the elbow-joint, of a year's standing. Excision on May 23. Doing well.

CASE VII.—St. Thomas's: Mr. South.—A woman, aged forty, in very fair health, the subject of disease of the knee-joint of three years' standing. Excision of the whole articulation on May 23. Death on July 9.

CASE VIII.—The Westminster: Mr. Holt.—A woman, aged twenty-three, in very feeble health. Disease of the knee-joint had followed an injury received seven weeks before admission. After six weeks' further treatment it became manifest that excision presented the only alternative to amputation, and it was accordingly performed on June 23. The curved incision was practiced, and about an inch of the femur and half an inch of the tibia sawn away, the patella not being interfered with. The cartilage was ulcerated on the posterior part of the outer condyle, and the corresponding surface of the tibia; the synovial membrane was pulpy and thickened, and an abscess, which communicated with the joint, extended up the back part of the femur. The tendons of each side were divided. She bore the operation remarkably well, and less constitutional disturbance followed than is usual after amputation. The limb is at present quite straight, and the wound nearly healed.

CASE IX.—The Westminster: Mr. Holt.—A very delicate boy, aged four. Admitted on account of necrosis of the femur, extending into the knee-joint, and of two years' duration. Three sinuses existed, two of which led down to diseased bone. Upon opening the joint by the usual curved incision, the cartilage of the external condyle was found wholly removed, together with that of the corresponding surface of the tibia. About three-fouths of an inch of the femur were sawn away, and half and inch of the tibia. In the extremity of the femur, as thus exposed, was a considerable cavity containing a sequestrum. The dead bone was removed, and the cavity gouged, leaving a mere circumferential shell. The patella being healthy was not interfered with, nor were the tendous divided, as the limb was easily brought into position. Considerable shock was felt at first, but afterward he became comfortable, and is now doing very well.

CASE X.—The Westminster: Mr. Holt.—A boy, aged seven, in a wretched state of health from disease of the hip-joint of two years' standing. Considerable swelling existed, and there were several sinuses leading to diseased bone. The femur was not dislocated, but forcibly retained in the acetabulum by false anchylosis. The head and neck of the femur were sawn away, and some necrosed bone from the acetabulum also removed.

CASE XI.—St. Bartholomew's: Mr. Coote.—A soldier, aged twenty-eight, in good health, admitted with disease of the elbow-joint of eight years' duration. A single long incision was practiced, and the articular extremities of the three bones removed. Recovered well.

CASE XII.—The Westminster: Mr. Hillman.—A boy, aged three, the subject of diseased elbow-joint of three months' duration, and consequent on injury. Carious bone was easily felt, and as his health was beginning to suffer, excision was determined on. A single long incision was practiced, and the extremities of the three bones were removed July 7. Doing well.

CASE XIII.—King's College: Mr. Fergusson.—A girl, aged fourteen, the subject of diseased elbow-joint from infancy. There was much chronic thickening about the joint and several open sores, but the child's health was still good. Anchylosis already existed. Mr. Fergusson cut out a wedge-shaped mass, which comprised the articular extremities of the three bones. Passive motion was commenced on the fourth day, and the process of healing advanced rapidly. Under treatment.

CASE XIV.—St. Mary's: Mr. Walton.—A strumous girl, aged fourteen, the subject from early childhood of chronic swelling of the knee. It had become painful only one month before admission. The tibia was partially dislocated backward and upward, and the knee bent at nearly a right angle. A splint was used for nearly a month, but without obtaining any benefit—the joint, on the contrary, having become more swollen, red, and painful. It was now determined to excise the joint. The horseshoe incision was adopted, and the patella and the articular ends of the femur and tibia were removed. The joint was found wholly disorganized. All seemed doing well for three days after the operation, when rigors set in, and were soon followed by delirium, jaundice, and extreme prostration. Abscesses formed on the wrists, and backs of hands. She died, with all the symptoms of pyæmia, on the tenth day. The wound had united to a considerable extent by first intention. The autopsy did not reveal anything of importance.

CASE XV.—University College: Mr. Erichsen.—A man, aged thirty-nine, in fair health, the subject of diseased elbow-joint, consequent on a blow three years before. Excision after the usual method on July 8. Doing well.

CASE XVI.—University College: Mr. Erichsen.—A healthy boy, aged seven, under care on account of diseased elbow-joint of three months' duration. Excision. Recovery.

CASE XVII.—King's College: Mr. Partridge.—A strumous-looking Creole, the subject of old-standing disease of the knee-joint. The joint was anchylosed at almost a right angle with the thigh, and he suffered extreme pain in the part. Resection was performed in the usual manner, and the leg put up in the swing-splint. Union slowly took place, and he was sent to the seaside for further benefit to his health. Recovered.

CASE XVIII.—King's College: Mr. Partridge.—A boy, aged thirteen, in fair health. The limb was useless from anchylosis in a bent position, and the part was moreover liable to frequent attacks of inflammation. An unsuccessful attempt had been made to straighten the limb under chloroform. In the resection the H-incision was adopted. About an inch of the femur was removed, a portion of the patella, and a thin slice of the tibia. The bones were found firmly anchylosed together. In sawing through the femur a small abscess was opened in its outer condyle, which had probably communicated with the joint. The operation was performed on June 13; all went on well, and the wound was almost healed on August 27, the bones being all but firm.

CASE XIX.—University College: Mr. Erichsen.—A boy, aged seven, the subject of disease of the hip-joint, of nearly three years' duration. The head of the femur was excised on January 7. After the operation the limb was kept in position by a bracket-splint. There was profuse discharge, and the boy was very low for some time. Abscesses subsequently formed, and second removal of carious bone was practiced on May 20. Since that he has slowly improved. Under treatment.

CASE XX.—King's College: Mr. Fergusson.—A man, aged thirty-four, for sixteen years the subject of stiff knee. The joint was wholly disorganized, and resection was accordingly determined on. The patient did well after the operation, and the splint was taken off at the end of six weeks. The operation was performed on May 2, and the man was discharged well on August 5.

CASE XXI.—King's College: Mr. Fergusson.—A strumous girl, aged twenty-one, for six months the subject of inflamed elbow-joint. Resection by the single long incision was performed on May 16, and the patient afterward did very well. She was discharged cured on July 29, having a very useful arm.

CASE XXII.—King's College: Mr. Fergusson.—A girl, aged ten, had been for several months under treatment on account of contracted knee. By means of splints the limb had been got almost straight. The joint, however, continued painful, and Mr. Fergusson determined to resect. The operation was performed in the usual manner, and the articular surfaces of the three bones were sawn away. The patient did remarkably well, and when, at the expiration of six weeks, the splint was removed, it was found that firm bony union had taken place. The patient was discharged on August 10, (two months after the operation,) with an excellent limb, the shortening being not more than half an inch.

CASE XXIII.—King's College: Mr. Bowman.—A boy, aged eleven, the subject of diseased hip-joint, of twenty months' standing. The femur was not dislocated, but its articular head was partially absorbed. It was resected, and, one edge of the acetabulum being carious, was gouged. He did well after the operation, and is now nearly recovered, having been able to be about on crutches for some months. The operation was on April 4. His health has greatly improved.

CASE XXIV.—King's College: Mr. Bowman.—A girl, aged five, for two years the subject of diseased hip-joint. In the resection the acetabulum was found to have had its borders broken down, and to contain soft gelatinous granulations, and pieces of loose bone. The horizontal ramus of the pubes was bare. The head of the femur had lost two-thirds of its substance. The child was placed in the hammock-swing (as designed by Mr. Heath) from the very first, and progressed remarkably well. The wound has now all but healed, and her general health is very good.

3. EXCISION OF THE KNEE-JOINT.—[The most perilous of the operations enumerated in the preceding report is undoubtedly that of excision of the ends of the bones constituting knee-joint—an operation which, although established years ago by Park and Moreau, has only very recently recovered from the opprobrious position into which it had fallen by the recklessness and incompetency of those who seized upon it soon after its first introduction, for the purpose of professional and public notoriety. To Mr. Fergusson, of London, is due the credit of its revival, and to Mr. Butcher, of Dublin, the profession is largely indebted for a complete exposition of the history of the operation, the circumstances under which it may be considered justifiable, the best methods of procedure, and the results so far ascertained. Mr. Butcher's instructive contributions may be found in the February number of the *Dublin Quarterly Journal of Medical Science;* and in the November number of the same valuable periodical he reports a case of his own, which so fully illustrates the great principles involved that our readers will excuse us for copying the account entire, notwithstanding its length.]

"Timothy Swift, aged twenty-seven years, a laborer, admitted into Mercer's Hospital April 8, 1857. *History.*—In February, 1854, the man was carrying a heavy sack of wheat; he slipped and fell to the ground; the weight crushed the limb. His left knee suffered severe stretching and contusion, which confined him to bed for nearly three months; after this the joint was stiff, and performed its motions very imperfectly, yet he returned to his employment. Shortly after, a dull pain lurked in it, greater at some times than at others, and some swelling remained. In about seven months after the primary accident he tripped in jumping over a ditch, and violently wrenched the joint a second time, after which he was obliged to remain in the house for several days. A few months passed over, and again he endeavored to earn his livelihood by employment; at this time frequent exacerbations of pain seized upon the part, and stiffness of the articulation increased. In this way he persisted in working on, but could not run or move the leg rapidly. Things thus progressed until about sixteen months before his admission to hospital, when he was violently thrown from a restive horse that he happened to be riding; the animal fell upon him heavily, bruising the left knee again; immediately after the accident the left leg and thigh swelled rapidly, and the patient was laid up, confined to bed, for about six months. After this he was treated by several medical men, but with no marked benefit, when he came to Dublin, in September, 1856, and was subjected in several hospitals to the most appropriate treatment. Not obtaining relief, he became discontented, and placed himself under the care of a quack doctor, who took all the money he possessed, and then dismissed him with the pleasing consciousness that he could not be cured. On the date already mentioned he sought my advice, and was admitted to hospital. Though his general health was not shattered to any alarming degree, there were many symptoms that awakened in my mind apprehensiveness of tampering any longer with a disease that was gradually, slowly, and surely undermining the healthy springs of life. The injurious consequences originating and set up from the local malady threatened, and that not far off, a more dangerous manifestation of their withering effects,—in few words, the management of the case required decision, promptitude, and judgment. The constitutional sympathy was evidenced by a rapid pulse, never below 100; by impaired digestion, a capricious appetite, frequent nausea, often vomiting. The body was but little wasted, yet on looking to the local changes, a very marked difference existed between the volume of the sound limb and that of the affected one. The right thigh measured in its circumference, at the widest part, 20½ inches, while the corresponding measurement in the affected limb was only 15 inches. The widest part of the calf of the sound limb measured 11½ inches, while that of the left was only 9 inches. The outline of the joint was lost, and its configuration spoiled; a puffiness of the soft parts filled up each sulcus around it, so that its walls and boundaries presented rather a cylindrical form. The patella was movable; there was free motion of the leg in every direction; great lateral mobility—that is, on the leg being pressed one way and the thigh to the opposite side, an unnatural amount of lateral motion was permitted, only to be explained by the destruction of the interior restraining ligaments of the joint; flexion and extension, to a certain extent, were permitted—indeed, extension was allowed nearly to the full, but flexion beyond a right angle induced the greatest torture; percussion at the heel elicited at once deep suffering and excruciating torture in the joint, so as to make the patient start with terror and alarm; there was no increased secretion within the joint, or marks of abscesses as having ever occurred. It was clear that the diagnosis pointed to thickening of the synovial membrane and ulceration of the cartilages, destruction of the head of the tibia to a greater extent than that of the condyles. All particulars considered, the case was by me considered perfectly suitable for excision.

"On the 15th of April, 1857, I proceeded to operate after the following manner, the patient being placed under the influence of chloroform. I adopted the H-incision, the cross-line passing beneath the patella; the flaps were with rapidity dissected back, and the shreds of the crucial ligaments spared by disease were divided, and next the lateral ligaments; in freeing the ligamentous attachments to the bones behind, the greatest precaution was adopted; all being separated to the extent required, I swept the knife around the tibia and the femur close to the attachment of the soft parts, and then took the saw bearing my name, and cut the bones from behind forward. It is necessary here to lay caution on the operator in using the saw; he should ever remember the altered position of the limb, to facilitate the protrusion of the ends of the bones, and according to the angle of elevation must the direction of the blade of the saw traverse. The simple rule I would lay down for the correct execution of the section is this: the blade of the saw must pass in a direction parallel with a line drawn in the transverse axis of the articulating surface; accordingly, this procedure was carried out; thus, when the limb is placed in a horizontal position, the one in which it is to be maintained for cure, the cut surfaces of the bones will be evenly together, no space will intervene between them behind or before; the wide surfaces oppose each other; all disposition to gliding one from the other is guarded against, and the most favorable circumstances are insured for intimate union. In the published records of cases it will appear that, in some instances, the surgeon has had to apply the saw a second and a third time to make the bones meet; if this be so, I am then warranted in enforcing my advice. By section planned after this method, the condyles of the femur, with their connecting osseous bond to the depth of a quarter of an inch, were removed, and a slice from the upper surface of the tibia, nearly three-quarters of an inch in thickness, was cut off. To warrant the removal of these parts I may just state that the incrustating cartilages of the condyles were entirely removed; the head of the tibia was similarly affected, and, in addition, deep pits were excavated by caries in each condyle, to the depth of a quarter of an inch. This being effected, all the thickened and diseased synovial membrane was clipped away, and the disorganized fatty mass below the patella; not a trace of the interarticular cartilage remained; the patella was coated with lymph beneath, and appeared to have struggled healthily from the disease around it; it was, therefore, suffered to remain. Thus, then, the accuracy of the diagnosis was established, and examination of the osseous surfaces pronounced them healthy. Three arteries, which bled rather freely, were next tied; the flaps at the transverse incision were brought together and maintained so by fine points of interrupted suture, and the lateral incisions were left open for the ready escape of blood and serum, the purging of the cut parts. The leg was with ease put into the straight position, and placed at once in the padded box-splint I had prepared for its reception; a splint was then laid over the anterior part of the thigh, and the tapes fastened, sustaining upward the hinged sides of the box; the foot was steadied by a footboard falling into the grooves within, and thus the leg was pressed upward so as to keep the divided osseous surfaces in contact; lint steeped in cold water was laid along the lateral incisions, and maintained accurately in position by the sides of the box when elevated. The chloroform acted admirably: though the man was at first thrown into some violence, yet after the lapse of a few seconds he was totally subdued, and slept unconsciously all through. Shortly after the anæsthetic was discontinued, he awoke quickly, and was unconscious of the operation having been performed. Thus, quite conscious, with the limb immovably adjusted, the man was removed from the operating theatre to bed, when he got a full draught of wine. Shortly after, he discharged his stomach, a not unfrequent consequence after the use of chloroform. This sickness should be watched for, anticipated on the part of the surgeon; and, after emesis has taken place, the pallor, coldness, and collapse can readily and entirely be removed by a full warm stimulant—none better than a tumbler of hot punch with an opiate in it. Such was administered in this case, and by me in many others, with the desired effect, persistent quietude of the stomach; to restore general warmth, heat should be applied to the limbs and body. In two hours after the operation reaction was fairly established, and the patient complained of pain; a general weeping took place from the external lateral incision, and also from the internal; the quantity was such as to call for interference to check it, and it was very simply done. The sides of the box were let down, and compresses of fine lint laid over the points from where the blood issued most copiously, and finger-pressure maintained them in their places: all disposition to hemorrhage ceased by a perseverance in this mode of treatment for little more than half an hour; then gently the hands were taken away; a few additional pads were laid over each compress, so that when the sides of the box were again elevated, a gentle and equable support was more forcibly afforded throughout. Thirty drops of tincture of opium was given every third hour; and I ordered ice to be placed in the mouth to quench thirst. 7 P.M. No return of bleeding, and pain not considerable; reaction fully developed. 9 P.M. Pain very trifling, and dozed away; gave a tumbler of punch and thirty drops of tincture of opium, after which he slept steadily, though before this he had slight spasms and tendency to startings of the thigh forward; however, this was controlled by the anterior splint and the full opiate, which now completed the sedative influence of the drug.

"April 16th. Had a good night and slept with tranquillity, and this morning is much refreshed; pulse 104; tongue furred, and thirst; urine freely passed in quantity, yet skin hot; limb lying in excellent position, and exempt from pain; took some tea and toast for breakfast. 3 o'clock P.M. Continuing to feel comfortable; opium to be continued. 9 o'clock P.M. Free from pain, no uneasiness whatever in the limb; thirst alleviated by effervescing draughts in combination with the opium, and by the placing of small morsels of ice frequently in the mouth.

"17th. Had an excellent night; pulse 100; skin still hot, but thirst diminished; full opiates every third hour throughout the day.

"19th. Going on most favorably; the patient slept all night, and took his toast and tea for breakfast with appetite; let down the sides of the splint, the external and internal alternately, to soak up the fluids discharged from the wounds, which was quite practicable without stirring the limb from its posterior support; the full opiates to be continued.

"20th. The patient slept all night without interruption, and this morning his pulse was down to 86: it is soft, yet not feeble; his tongue is clean, the thirst nearly gone, and his bowels gently moved, and urine passes in full complement; he took his breakfast with appetite. I let down the sides of the box and soaked up purulent matter, which freely escaped from the wounds; placed dry linen over the pads on either side, but did not lift or disturb it from the surface upon which it rested.

"29th. Since last report everything has progressed in the most favorable way; a slight fullness was perceptible toward the inner side of the lower third of the thigh; this, however, was effectually controlled after fifty-six hours' pressure of well-applied graduated compresses, gently directing the discharge from behind forward. The transverse wound is now quite healed, and the lateral ones discharging pus freely, which I cautiously removed each day, as already specified, without lifting the limb.

"May 5th. This day, for the first time since the operation, I lifted the limb from the box, twenty days after the operation, the anterior splint being firmly held to the limb by assistants, or, rather, the limb up to it, and renewed all the dressings. No soreness or excoriation of the posterior surface of the buttock, thigh, or back; neither was there any pointing of matter backward, the lateral incisions affording a ready outlet for it as quickly as it might be secreted.

"May 6th. Discharge greatly diminished; wounds quickly closing in; no pain; sleeps without an opiate now, and no tendency whatever to spasm; he eats and drinks freely. Removed to a fresh bed.

"June 1st. Ever since last report gradually and steadily proceeding. The discharge has become very much diminished, and the lateral wounds contracted considerably; pressure on the patella gives no pain, and but little pus is forced out by pressure upon it; the leg and thigh are becoming rigidly connected; and pressure on the heel does not in the least degree elicit pain—a sufficient evidence that the change brought about at their junction is a healthy process.

"It is unnecessary to continue the daily report: both the careful dressing of the part, the mode of treatment pursued upon the limb, and the dietetic rules prescribed continued to be the same, with but little alteration, up to July 2d. At this period a small abscess formed just below the patella; from it matter could be pressed out through the external lateral wound; but I cut short its route, and passed a knife into the abscess perpendicular to the surface; this allowed immediate exit to the contents, and at once almost the propriety of the measure was borne testimony to by the quick consolidation of the parts around, and the total dispersion of œdema and swelling. Now so healthily did the repair go on, and so effectually between the divided bones, that the patient could lift the limb *en masse* from the bed without the least fear. No yielding of the parts was consequent upon this trial—nay, more, on taking the limb between the hands and moving the leg and thigh in a contrary direction, antero-posteriorly or laterally, not the least motion was produced between them—an efficient test of the stoutness and integrity of the combining medium, the bond of union. Shortly after this an abscess and sinus formed above the patella, and to the inner side, over the sheath of the vessels in Hunter's canal; the contents from it could be gently pressed toward the internal lateral wound, and thus a ready outlet afforded for its discharge; pads, compresses, and well-applied, broad, adhesive straps obliterated it in a short time with accuracy and precision. The thickening of the parts around, particularly in the vicinity of the patella, was quickly reduced by suitable bandaging from the toes toward the knee, and from the groin downward, both meeting around and supporting the newly-applied and engrafted parts. After each dressing of the limb it was again steadily fixed in my fracture-box.

"The review of this case from first to last is most pleasing: from the beginning to the end there was never any alarming symptom. I attribute the favorable issue to the following causes:—

"1st. The judicious selection of the case; for, though the joint was irretrievably destroyed past all hopes of repair, yet the bones were not diseased beyond their articulating surfaces—therefore too large a quantity had not to be cut away.

"2d. The operation was expeditiously done under chloroform; therefore comparatively little shock.

"3d. There was no hemorrhage; no persistent weeping from the wound permitted; therefore but little exhaustion.

"4th. The wound being dressed in a special manner, the limb was at once placed in the box, and the adjusted bones restrained from the slightest deviation forward, backward, inward, or outward. Moreover, by the adaptation of the footboard the leg was kept fairly pressed up against the thigh, which in its turn was sustained in a contrary direction by the weight of the trunk, the proper axis being secured by the long arm of the splint resting parallel to and against the body. Now it should be borne in mind that this portion of the splint was not as yet lashed to the trunk by the web-girth, because during all this mechanical appliance the patient was insensible, under the influence of chloroform, and any pressure on the chest, so as to interfere with respiration during this tranquil sleep, not only would have been injudicious, but highly dangerous.

"5th. The position of the wounds, each lateral one being placed well back toward the popliteal space, thus readily allowing a free exit for all secretions, and effectually guarding against all pouching in the ham.

"6th. The quietude, the repose in which the limb was suffered to remain for several days after the operation,—the long period of three weeks having elapsed before it was gently and steadily lifted, after the manner described, from its bed; though prior to this time on several occasions the discharge was readily soaked up, without the slightest disturbance of the member, owing to the construction of the box.

"7th. The full exhibition of opium during those days when wicked irritation might have been anticipated; the due exhibition of stimulants and nutritive diet, from the moment that the stomach would tolerate them at all to the termination of the cure.

"The condition of the man at present (11th September) is all that can be desired: long since the box has been laid aside, and the limb is steadied by the application of a splint behind, as in my former case; the bones seem to be grown into each other; there is not the slightest yielding or motion between; it is a permanent union, and resists shocks; the most forcible percussion at the heel neither elicits pain nor motion; the patient is able to rotate the limb from the hip inward and outward, flex and extend it with ease and confidence. There is a slight discharge from the wound on the inner side still, but this I have rather solicited, keeping it open as a drain by which any secretion, either of pus or serum, might readily escape. I have not permitted the man yet to walk upon the limb, though I have no doubt he could do so, and indeed he feels that he could do so if he tried; he lies upon the outside of the bed with his clothes on, and in a few days he will be walking about. The motions of the hip and ankle-joints remain as flexible as before the operation. The subject of these remarks is now as robust and as strong-looking as any man to be seen, and on contrasting the limbs as they rest side by side the amount of shortening is but very trivial. The degree of emaciation is not so characteristic now as before the operation. On the whole, the case is one with which I feel very deeply satisfied."

4. OPERATION FOR IMPERFORATE RECTUM NOT IMPRACTICABLE. By *C. E. Buckingham, M.D.*—William, the son of William Lund, was born on the 6th of December, 1851, and on the morning of the following day the nurse reported that there was an obstructed anus. On examination, the cleft of the nates was found sufficiently marked, but there was no evidence of an anus, either by protrusion or discoloration. During the night there had been occasionally bilious vomiting, and latterly straining, as if to evacuate the bowels. There had been no discharge of urine. Had taken no food of consequence, but had tried to nurse. The countenance looked badly, and there was lividity about the mouth and eyes. Was somewhat stupid; did not cry, but was constantly moaning.

Operation thirty-two and a half hours after birth, with the assistance of Dr. Henry Osgood Stone. The child was held upon the lap of the nurse, its nates resting over the right knee, and the knees raised as for lithotomy. I made an incision in the centre of the cleft of the nates, from the scrotum to the coccyx, and crossed this with another, at right angles, from the tuberosity of one ischium to the other. The dissection was carried on with a sharp-pointed straight bistoury, backward, and a little to the left, for two inches. No evidence of the neighborhood of the rectum being obtained with the finger, I passed a hydrocele trocar into the wound, in the same direction, a half-inch further. On withdrawing it, meconium was found upon it. The wound was then enlarged with the knife, and a female catheter was introduced, through which an enema of warm water was administered. There was immediately a fair discharge of meconium, and a slight discharge of urine.

The child cried but little, and the whole loss of blood was not much more than two drachms. A few spoonfuls of milk and water were given, and it was dressed in the usual manner, no application being made to the wound. Half an hour later the moaning had ceased, the child looked brighter, and there was a profuse dejection.

5 P.M. Has had two full evacuations, but has not vomited nor passed urine. At 5 P.M., on the 8th, I introduced a sponge-tent two and a half inches, with some little difficulty. During the attempt at introduction, the efforts of the child to evacuate the bowels produced an audible passage of air through the penis, which was rendered visible by the spattering of urine, and perceptible to the hand, which was laid above the pubes.

Dec. 9th. Removed the tent, immediately after which he passed a large amount of almost colorless urine in a jerking stream. No passage, either fluid or solid, by the anus at the time.

I have the regular reports of each visit, but select only such as are of particular consequence.

Dec. 14th. Tried unsuccessfully to introduce a bougie of more than one-fourth of an inch in diameter. Umbilical cord has not yet separated.

16th. Introduced a female catheter with difficulty.

25th. Free dejection. Passed a bougie of ebony, seven-sixteenths of an inch in diameter.

27th. In pain all night. Tumor in left side just over short ribs, size of a small walnut. Nurse says she discovered it last night.

29th. Tumor increasing in size. Passed bougie of 25th again with ease. Some bloody pus followed it.

Jan. 1st, 1852. Opened tumor, which discharged an ounce of pus.

7th. For several days the left side of the scrotum has been swelling.

13th. Opened small abscess in front of scrotum. Child weighs 9½ pounds, an increase of 2½ pounds since birth.

22d. Bougie has not been introduced since the 16th. Has two dejections daily, and sometimes more. The scrotal abscess is well; there has been a slight gathering again on the left side, which broke yesterday and does not discharge to-day.

Feb. 13th. No bougie since Jan. 16th. Three dejections. Weighs 11½ pounds.

March 11th. Gains daily. Nurses well. Bowels open freely every day, without medicine. No bougie since Jan. 16th. Has gained another pound.

Soon after this last report, the child left town for Gardiner, Me., and returned on the 28th of May. Saw it that afternoon. Looks well, and is fat and hearty. About the 1st of May discharged urine and fæces mixed, by urethra; but has not since. For several days last week had diarrhœa, which stopped on the 21st. No instrument has been passed into the anus, which is red and shining about its edges, and bled a little on separating the nates.

Aug. 5th. Has six teeth. For several weeks has had diarrhœa, and fæcal matter passes by the urethra as much as by the anus.

In the fall the family removed to Malden.

Oct. 21st, 1857. Saw Mrs. Lund, the mother, at 36 Leverett Street. She informs me that her boy, upon whom I operated, is still living, and is generally in good health. He occasionally has pain in the pubic region, but she considers him well. There is, however, at times, difficult micturation. The family still reside at Malden.

The above case is given, because it was stated, as appears by the records, at the Boston Society for Medical Improvement, as the belief of one gentleman, "that in the present state of the art it is better that a child born with either of these imperfections (*of anus or rectum*) should die without this operation, although it must occasionally be performed in deference to established opinion." The question may be asked if my case is not one of those exceptions which are said "to prove the rule." If it be so, then the rule is one of those which should be honored in the breach. An *ex cathedrâ* statement—as that from an officer of the Massachusetts Medical College is, and ought to be—may have an unfortunate effect upon many a timid physician, who dares not think for himself, and who would hesitate to ask the aid of one who denounced the operation. If there has been one successful case, which the profession have not known, it it is very probable that in the case-books of other private practitioners there are other such. It is very likely that they have not been brought to light because physicians have had no reason to suppose them peculiarly fatal until now.

The case reported by Mr. Jones, at the Suffolk District Medical Society, has been spoken of as if it were not a fair case of imperforate rectum, because the sphincter contracted upon the finger. If the child had died without a *post-mortem*, would any one have questioned its being a *bonâ fide* case? The recovery is the only evidence that there was merely a septum across the gut.

The case which I have reported is one of imperforate anus, absence of the lower part of the rectum, and communication with the bladder.

So far as my own reading extends, I do not find any great distinction made by writers between imperforate anus and imperforate rectum. The names do not convey a just idea of the malformations, particularly if, by the name, one is to decide upon the propriety of attempting to save life.

In the *Edinburgh Monthly Medical Journal* for January, 1857, which must have been seen by a large number of the members of the profession in this city, is a case precisely like the above. The patient is still living, at the age of thirty-six years, and in perfect health.

Samuel Cooper not only thinks the operation justifiable, but says, "It is the surgeon's duty to do everything in his power to afford relief," and then goes on to describe the operation. He follows with the statement that "by such proceedings many infants have been preserved," in some of whom incisions two inches and more have been made, and alludes to cases by Wolff, Hildamus, La Motte, Roonhuysen, Hutchison, Benj. Bell, and Miller.

It is certainly remarkable that these cases should have escaped the notice of the gentlemen connected with the only institution for instruction in medicine and surgery in active operation in this city at the present time; and it will be equally remarkable if the officers of other schools should arrive at the same conclusions as they have respecting the operation.—*Boston Med. and Surg. Journal*, Nov. 26, 1857.

5. LACERATION OF THE URETHRA FROM MUSCULAR CONTRACTION, WITHOUT EXTRAVASATION OF URINE. *Reported from the Practice of Dr. J. C. Hutchison, in the Brooklyn City Hospital, by R. O. Butler, M.D.*—W. P., seaman, aged twenty-six years; native of United States; was admitted into the hospital, March 25th, 1857, at three P.M. Twenty-three hours before admission he was struck over the region of the left trochanter major by the end of a sugar-hogshead which was being lowered into the hold of a vessel. He was unable to walk after the injury, but could stand on both legs. On the following day, however, he was unable to stand, in consequence of the pain about the left hip-joint. Three or four hours after the accident he attempted to evacuate his bladder, but could not do so, and he observed at this time that the orifice of the urethra was closed with coagulated blood.

At the time of admission he was suffering considerably from retention of urine, which had existed since the occurrence of the accident; he also had a good deal of contusion and pain in the neighborhood of the left hip-joint, which prevented him from turning in bed. Moving the joint increased the pain greatly. *There was no pain, contusion, or swelling then, or subsequently, about the perinæum, scrotum, or penis.* The catheter, introduced by the House Physician, (my hand was still disabled,) glided out of the urethra at the membranous portion to the left, and passed backward, with very slight pressure, so far that there was barely enough of the instrument outside of the meatus to hold between the thumb and fore-finger. On introducing the finger into the rectum the instrument was felt immediately in front of the gut, nothing intervening apparently but the coats of the bowel. The catheter was passed into the bladder after much difficulty, and the urine drawn off.

On the following morning (26th) he was suffering from retention of urine, and

Dr. Krackowitzer, who was visiting the medical wards before the arrival of Dr. Hutchison, introduced the catheter. The instrument several times glided into the false passage, and was detected by the finger in the rectum, immediately in contact with the coats of the bowel. Dr. Hutchison passed an instrument on the same day, and also observed the false passage. No extravasation of urine occurred; the catheter was retained in the bladder eight days without local pain or constitutional disturbance. After its removal the urine was discharged spontaneously, and for three or four days without scalding, but it contained considerable mucus. He had now recovered from the effects of the contusion over the hip-joint, walked about the ward, and felt quite well; his urine, however, could not be retained as long as usual—its discharge was attended with scalding, and it contained mucus in considerable quantity, which adhered to the bottom of the vessel when turned up. He used a copaiba mixture and mucilaginous drinks, but no material change took place, and he passed into the hands of Dr. Minor, whose term of service commenced May 1st.

The irritability of the bladder was now considerable. He had to discharge his urine several times during the night, and was sometimes unable to sleep on account of pain in the bladder. The urine contained pus and mucus. Pariera brava, liquor potassæ, copaiba, etc. were used successively without benefit, and on June 13th an injection of one ounce of a solution containing ten grains of nitrate of silver was thrown into the bladder. It produced considerable pain for a time, which was followed by a marked improvement. He did not get up to pass urine for three or four nights, and the quantity of mucus and pus was greatly diminished.

Six days subsequently the injection was repeated, and was followed by the same benefit as before.

June 30th. Urine passes in pretty good stream, unattended with pain or scalding, unless he walks about too much. The deposit of pus and mucus is one-half less than it was before the injections. Being anxious to visit his friends, he left the hospital, against advice, in July.

The prominent symptoms presented by the preceding case—viz. a discharge of blood from the urethra, retention of urine, great difficulty in introducing an instrument, and the existence of a false passage, observed immediately after an accident—evince, I think very satisfactorily, the nature of the injury to the urethra. It is especially interesting in two particulars—1st. For having been unattended by extravasation of urine, which must have been owing to the valvular nature of the laceration. 2d. The rupture was not caused by direct external violence, but was probably produced, as suggested by Dr. Isaacs, by the violent contraction of the muscles of Guthrie and Wilson, and, perhaps, of the transversus perinei, which took place when the accident occurred.—*New York Journal of Medicine*, Nov. 1856.

6. LIGATURE OF THE DORSALIS PENIS ARTERY FOR HYPERTROPHY OF THE PREPUCE.—D. D., a native of Martinique, aged thirty years, was admitted into the hospital in December, 1856, suffering with chancre, accompanied with con-considerable hypertrophy of the prepuce. The chancre healed under appropriate treatment, but the enlargement of the prepuce remained.

Being anxious to have the size of the organ reduced to its normal dimensions, Dr. Hutchison tied the dorsalis penis artery, for the purpose of producing atrophy of the part by diminishing its vascular supply—that being the principal vessel through which the prepuce is supplied with blood. The ligature came away on the fourth day, and no untoward symptom occurred, except a want of action in the wound, which rendered stimulating dressings necessary. Pressure was applied to the prepuce by means of collodion, containing three grains of iodine to the ounce, which was painted over its surface. He left the hospital June 7th, the hypertrophy being somewhat diminished.

Although ligating the dorsalis penis artery in the above case was not followed by complete relief, the result indicated that the operation may in some cases prove valuable. The suggestion is therefore deemed worthy of being recorded. The arrest of abnormal developments about the face, erectile tumors of the orbit, etc., by diminishing their vascular supply, is well known. I am not aware that the above operation has ever before been performed.—*Ibid.*

7. Removal of Warts by Chromic Acid. By *I. L. Crawcour, M.D., etc.*—In a late number of the *Medical Times and Gazette*, appeared some experiments by Mr. Marshall, on the use of chromic acid. Two cases have recently come under my own observation, and the results have been very satisfactory. A gentleman called on me about three months ago, complaining of a wart on the top of his head: it was about the size of a twenty-five cent piece, and the extremity was rough and split vertically. He had suffered from it for several years, and it gave him a great deal of trouble, bleeding whenever he combed his head. I painted it thoroughly, by means of a glass brush, with a saturated solution of chromic acid. The remedy produced little pain, the surface of the wart instantly blackened, and twelve days afterward a scab fell off, leaving a clear reddish surface underneath, and perfectly smooth. I examined this gentleman's head a few days ago; the skin is now perfectly healthy, and hair is growing on it. About two weeks ago, another case presented itself, of a similar character. I applied the same remedy. The scab, however, has not yet separated, but I fully expect the result to be identical with the former. The advantage of this caustic is, that it leaves no scar, and does not destroy the hair-bulbs when applied to the scalp. Its influence is undoubtedly owing to the readiness with which it parts with its oxygen, yielding half of its oxygen when applied to organic substances, passing to the state of green sesquioxide of chromium—this being the most powerful oxydizer we possess. Its preparation is very simple. Make a saturated solution of bichromate of potash, and add strong sulphuric acid as long as any precipitate of chromic acid falls; pour off the supernatant liquor, and dry the residue on a tile or brick. I prefer filtering through a glass funnel partially filled with asbestus. In preparing it, caution must be used not to allow any organic substance, as paper or wood, to come in contact with it, as instant decomposition ensues. The solution I employ is one part chromic acid and one part water.—*New Orleans Medical News*, Nov. 1837.

8. LIGATURING ONE OF THE COMMON CAROTID ARTERIES FOR THE CURE OF EPILEPSY. By *C. Angell*, of Pittsburg, Indiana.—[We quote the following cases, not for the purpose of encouraging the operation which they detail, but only to illustrate its results.]

Have we any known remedy for the cure of epilepsy? Without attempting to explain the nature, cause, or phenomena of this disease, I will present the result of two operations of tying the right common carotid for the cure of this disease.

CASE I.—The first operation was performed on the 2d day of July, 1857, on Wm. Brackus, of this place. He was twenty years of age, short, not over five feet in height, heavy set, large head, short neck, full habit, sanguine temperament, right arm and hand deformed, idiotic in most respects. Has been having fits three or four years, seldom at first, but gradually growing in frequency and severity. After having had a variety of remedies tried without any good result, I proposed tying one of the common corotids, thinking that by permanently lessening the amount of blood passing to the brain that it might cure him. After having had fifteen or twenty fits in the forepart of the day, he was put under the influence of chloroform, and I then placed a ligature on the right common carotid, about one inch below the bifurcation of the external and internal. The operation was performed without any difficulty.

The next day,—pulse 120; complains of difficulty in swallowing; talks with difficulty; complains of no pain; his mind about as it was before the operation; left side partially paralyzed.

Treatment: solution of salts and tartar emetic, as a cathartic and sedative. They operated well; wound covered with oil-silk, and kept constantly wet with ice water; gave nothing to eat but water-gruel and rice-water.

Without describing each particular day, I may say that he continued about as I have described him to-day: paralysis of the left arm and leg complete; still talks and swallows with difficulty; not much swelling about the wound and neck.

He remained in this condition until the night of the seventh day from the operation, when he died in a comatose condition. He never had any symptoms of fits after the operation.

An examination of the neck the next morning after he died, showed no signs of inflammation; the parts all looked healthy and natural. I removed a section of the artery, two inches in length, above and below the ligature; coagulation was quite firm in artery. The family think he would have died as soon as he did if he had not been operated upon. I do not think so.

CASE II.—The second operation was performed on the 8th of July, the same day on which the first patient died. This was on J. Bostick, of Brookston. He is forty years of age, naturally good constitution, full habit, sanguine temperament. Has had fits for seven years, but not so often or so hard, until the last three years; has them now almost every day, so that he is incapacitated from doing anything at all; his mind is also destroyed. He has been under treatment by a great many practitioners, both regular and irregular, but without benefit.

I performed the operation in the same way as in the former case. He soon came from under the influence of the chloroform, said he had suffered no pain, talked without any difficulty.

July 8. Says he feels well to-day; pulse 80; no unfavorable symptoms; swallows well, mind clear, talks well. I bled him this morning very freely, and put him on tartar emetic and salts, as in the other case. He has continued to get along well, pulse never over 80. He would every few days wake up bewildered or scared, this being the only symptom of returning fits until the 30th, when he had one so severe as to deprive him of sensibility; the next day had one about the same. He says they came on him different from what they did before. Before this he had no warning or premonitory symptoms of their approach; but now he feels a sensation of dizziness of some moments' duration, long enough for him to lie down before they came on.

On the 17th of August he had a slight fit, and on the 5th of September another, making in all four since the operation: it is now the 18th of September.

The ligature came away on the twenty-second day; the wound is all healed up. He is going about attending to his business. He says he feels better than he has done for three years.

His family, and those that are well acquainted with him, all say that they can see a marked change in him,—in his actions, countenance, and more particularly in his mind.

What will be the result I cannot tell. He now has the appearance of being benefited; how long this will continue I cannot determine. Whether the bettering of his condition is owing to the operation, or to the treatment, I cannot say.—*North-Western Medical and Surgical Journal*, Oct. 1857.

9. Novel Method of Extracting a Foreign Body from the Œsophagus. By *David Rice, M.D.*—Mrs. Field, a lady, aged seventy, while eating chicken-soup, accidentally swallowed a piece of bone the size of an American quarter of a dollar cut into a triangular form. The bone lodged in the œsophagus, about two inches below the top of the sternum. Thinking that it might fall into the stomach, she neglected to apply for surgical aid until the fifth day after the accident. In the meantime, she had swallowed neither food nor drink, both regurgitating into the mouth with every attempt.

I was called the fifth day, to try to remove the bone by surgical means. My first attempt was with a piece of whalebone, the extremity being perforated with numerous small holes, into which were fastened a dozen or more loops, about an inch long, made with small linen twist.

With this contrivance I failed, after many patient trials. I could readily reach the bone, but the loops did not fasten to any point of its angular form with sufficient permanency to enable me to extract it. I could even pass the piece of whalebone beyond the foreign body, and ascertained that it rested upon the posterior side of the œsophagus, standing perpendicularly, with two of its corners fastened into the gullet.

I finally took a piece of dry sponge about an inch long, and of such a shape, when dry, as to fill one-half of the œsophagus. This I tied to the extremity of my whalebone-sound. Turning back the head of the patient, I passed it down the œsophagus, *in a dry state*, as rapidly as I dared to do, until I was certain it had passed beyond the bone. I then introduced a little fluid into the mouth, which quickly reached the dry sponge, enlarging it to twice its natural size, completely filling the gullet. I drew it out, and with it came the bone, much to my gratification and the patient's relief.—*Boston Medical and Surgical Journal*, Dec. 3, 1857.

10. FALL ON THE EDGE OF A BROKEN DISH WOUNDING THE NECK AND SEVERING THE COMMON CAROTID ARTERY; LIGATURE OF BOTH ENDS, WITH RECOVERY. By *George D. Gibb, M.D., M.A., L.R.C.S.I., etc.*—Miss Q——, forty-five years of age, of robust frame, and enjoying excellent health, met with the following accident on August 29th, about noon:—while carrying in her hands a brown earthenware dish, containing an uncooked pudding, the point of her foot caught in a hole in the carpet, she stumbled, the dish fell to the floor, breaking into pieces, and she fell forward, striking the right side of her neck against a piece of the broken dish, which entered the skin below the level of the upper part of the thyroid cartilage, and wounded the common carotid artery. The right temple was also struck and bruised. On receiving these injuries she called out, was raised, and complained only of her right eye; her neck, she said, was nothing; but her father, seeing the blood pouring from the neck in a large stream, had the presence of mind to hold the sides of the wound together till assistance was procured. In about fifteen minutes it arrived, but during that short time, notwithstanding the precaution employed, upward of two pints of arterial blood were lost. The wound, about an inch long, transverse in direction, was closed, the neck bound up, and the hemorrhage ceased. She remained quiet till between three and four o'clock, when she suddenly vomited, and a gush of blood poured from the wound, which now commenced to flow in a large stream; the bleeding was again controlled by her father, while waiting for assistance, with the loss, however, of four pints of this vital fluid.

Mr. Whidborne, who had been sent for, arrived with Mr. Statham at half-past four P.M. The latter gentleman found a very free arterial gush from the wound; the finger introduced into the wound could be directed in almost any course in a ragged cavity, perhaps three inches in length and breadth, and from one to two inches deep. He enlarged the wound in the course of the carotid artery, removing pieces of mastoid muscle and clots of blood, and discovered the source of the hemorrhage to be the main trunk itself. At this time he found it impossible to wholly compress the bleeding vessel and control the hemorrhage without further assistance. I accordingly was sent for, and managed, with the ends of my two fore-fingers, to completely check the escape of blood. The dissection was proceeded with, the wound still further enlarged, and the trunk of the common carotid was got hold of and tied, immediately controlling all bleeding; this was effected about an inch and a half above the sterno-clavicular articulation. On tracing the sheath further upward and slitting it up, the vessel was found to have been torn across in an irregular and jagged manner, the upper end having retracted for upward of an inch and a half. This was only got hold of by further dissection and enlarging the wound upward, when its presence was revealed by a gush of blood, and it was immediately secured within its sheath. The wound was left open, and a piece of cold wet lint applied over it. As much blood was lost up to the time of my arrival as before Mr. Whidborne came—namely, four pints, making not less than eight pints altogether since the act of vomiting.

The patient was now very cold and pulseless, the breathing was almost indistinct, and she lay motionless. Small quantities of stimulants were given at intervals, such as brandy and beef-tea. At ten P.M. her pulse was 96, very feeble and weak; reaction set in after midnight, and next morning the pulse was 100. She was frequently troubled with retching, but the stimulants were persevered with.

The case subsequently did well; the treatment consisting mainly in the plentiful administration of beef-tea and other nutrient fluids, by the mouth and anus, the free use of alcoholic stimulants and opiates. There was but little hemorrhage; the upper ligature came away on the thirty-fifth, and the lower on the forty-fifth day.

She was now convalescent, the wound having closed. Her recovery was perfect, and she left for the country on the 28th of October.

During her illness, large quantities of wine and brandy were given, with plenty of the most nourishing diet, her strength being supported and sustained in every possible way. These were aided by a good constitution and much patient endurance on the part of the patient herself. She had no head-symptoms, but felt her right arm stiffer and weaker than the left; the pulse, moreover, throughout to the present time, has been almost double the volume of the left.

*Remarks.*—Any one familiar with the phenomena of hemorrhage is well aware that so large an amount of blood cannot escape, in so short a space of time as occurred in this patient, from the wound of a small arterial trunk. Let the wound be never so small, or even allow that the hemorrhage is easily controlled by simple closure of the wound, still the evidence stands before us that upward of two pints of blood had flown very rapidly from the wound. This points very clearly to the fact of a main arterial trunk being wounded; and the first duty of the surgeon should be boldly to ascertain the true source of the bleeding. This being done, his course is then clear as to any ulterior proceeding. Without casting reflection upon any person, it may be truly stated that an oversight was committed in the early treatment of this case, by remaining contented with the arrest of bleeding by simple closure of the wound with adhesive plaster—the knowledge of the large amount of blood already rapidly lost we will suppose borne in mind. The act of coughing, some hours after, reopened the wound; and although assistance was speedily procured, about a gallon of blood had escaped before the proper steps of tying the severed vessel were completed. This might have been prevented had the bleeding vessel been secured at first. The wonder is that the patient rallied from her extremely exhausted condition; but she did do so, and made a most excellent recovery.—*Lancet*, November 14, 1857.

11. A Large Uterine Polypus Removed by the Curved Ecraseur with Double Action. *Reported to the Medical Society of London by Dr. Savage.*—A woman, fifty years of age, who had been suffering from frequent uterine hemorrhage during the last two years, was admitted into the Samaritan Hospital ten days ago, under Dr. Savage's care. Many examinations had been made elsewhere previously by various surgeons; but a polypus, if suspected, until the day before her admission was probably quite out of reach. A swelling could even now be scarcely made out by the finger introduced far into the os uteri. Sponge-tents were introduced daily. On the third day the tumor became more distinct, and then rapidly distended through the dilated os into the vagina. On the fourth day it could be felt in size and shape like a large jargonel pear, (its neck not much less than its body,) extending into the uterus, to be attached somewhere toward its back part. On the fifth day the polypus was laid hold of by a pair of ring-forceps, the looped chain of the écraseur being passed over the handle of the forceps, slipped up, and was drawn tight, precisely as the cord in the ordinary operation by ligature, and the tumor was brought away without pain or hemorrhage. Dr. Savage observed that the unwieldy look of the instrument was suggestive of much pain and difficulty; but its curve fell into the hollow of the sacrum with the utmost facility, and its point as readily passed into the uterus as high as he thought necessary. As the chain is flexible only on one side, much careful manipulation was required before it could be got where he thought it ought to be. Before working the handle which tightened the chain, the single forefinger readily ascertained that nothing improper was included. The handle was worked at half-minute intervals as soon as decided resistance showed that contraction had commenced. The tumor came away in six minutes. From first to last the patient said she felt no pain whatever. The hemorrhage has not reappeared. Dr. Savage said he had heard of two cases of polypus thus treated in this country, but believed they had not been recorded. He had several times seen M. Chassaignac remove parts highly vascular, with his écraseur, without the least hemorrhage, and thought, as the plan he had adopted in this case was equally safe as the ligature, without any of its obvious annoyances, he would add his testimony to its value through the Medical Society. Dr. Savage strongly recommended an instrument with the double action, the finishing improvement of the inventor, M. Matthieu.—*Ibid.*, Nov. 21, 1857.

12. THE PRESENT STATE OF SURGICAL SCIENCE IN REFERENCE TO CANCER AND ITS TREATMENT. (Read before the Medical Society of London by *Mr. Hird.*)—It was not his intention, the author observed, to enter into a detailed report of individual cases treated by the escharotic applications which of late have been revived by many members of the profession besides himself, but to inquire whether recent experience has shown that we can control the progress of this destructive disease with more certainty, and on sounder surgical principles, than our predecessors were enabled to do. The questions whether carcinoma ever originates and continues as a local disease; whether it ever spontaneously disappears without the interference of art; whether a tumor, originally innocent in its character, is susceptible of cancerous transformation; whether it possesses anatomical, chemical, physiological, and pathological characters, by which we can infallibly distinguish it from other growths, which we are unable to do by the senses of sight and touch; whether, even supposing the constitution to be affected, the ablation of the local disease may not arrest or retard its fatal course; whether removal ever accelerates it by bringing into activity a force which previously had lain dormant in the system; whether we possess remedial agents by which we can retard or resolve the disease, he considers, embrace subjects of vital consequence, on the solution of which the life of the patient, the truths of science, and the reputation of the surgeon depend. In his observations on the forms of cancer, the author included the epithelial variety along with the encephaloid, scirrhus, and colloid, and objected to its removal from the cancerous group, although it has less tendency to contaminate the lymphatic glands and the system generally than the other three forms of cancer, and differs slightly in its histological elements. After briefly reviewing the important subjects embraced in the origin and development of cancer, in which the preceding questions were answered so far as the present state of science would admit, he proceeded to discuss the treatment. In this section the author dwelt on the means of arresting cancerous growths by medicinal agents, and contrasted the comparative advantages of removing the local disease by caustics and the knife. In reference to medicines, the author believed that if any remedy possessed a power of retarding the progress of the disease, it was arsenic, which he administered with cod-liver oil, and has not found the latter objectionable on the ground of animal oils tending to encourage the fatty matters in the system, on which cancerous formations are supposed to feed. Arsenic, as a preventive of secondary formations after removal of the local disease, he (Mr. Hird) had great confidence in, and in combination with iodine it was strongly recommended by the late Dr. Anthony Todd Thompson; Dr. Copland and the late Mr. Hill witnessed great advantages from its use. The great question of removal by the knife or by caustics concluded the paper. Caustics the author had found more tedious in their operation and more painful than the knife, and occasionally failed to enucleate the tumor, and he has no faith in a supposed physiological action on the constitution through the part treated, by which it has been stated, without evidence to justify the statement, and, in opposition to his own experience, the disposition to return after removal is less than when the part has been extirpated by the knife. He has seen six cases in which the disease returned after removal by caustics, and has read and heard of many more. Nevertheless, there are cases in which caustics offer greater advantages than the knife. In certain localities, and in particular conditions of the tumor, and where loss of blood, or the dread of a cutting operation, would greatly depress the patient. Early operation the author strongly advised, as affording the only hope from surgery in scirrhus, colloid, and epithelioma. In encephaloid he did not advise an operation.—*Ibid.*, Oct. 17, 1857.

